# The complete chloroplast genome sequence of *Paris nitida* G.W.Hu, Z.Wang & Q.F.Wang 2017 (Melanthiaceae), an endemic herb in China

**DOI:** 10.1080/23802359.2025.2519214

**Published:** 2025-06-17

**Authors:** Jinghan Wang, Jiwu Cao, Junsheng Liang, Wei Guo

**Affiliations:** ^a^ College of Forestry, Soil and Water Conservation, Central South University of Forestry and Technology, Changsha, P. R. China; ^b^ Hunan Academy of Forestry, Changsha, P. R. China; ^c^ Taishan Academy of Forestry Sciences, Taian, P. R. China

**Keywords:** *Paris nitida*, chloroplast genome, illumina sequencing, melanthiaceae

## Abstract

*Paris nitida* G.W.Hu, Z.Wang & Q.F.Wang 2017 (Melanthiaceae) is a perennial herb that was first identified in China in 2017. In this study, we determined the circular chloroplast genome of *P. nitida* through *de novo* assembly. The 164,734 bp genome exhibits an AT content of 62.89%. We annotated 131 genes, including 36 tRNA genes, eight rRNA genes, and 87 protein-coding genes. Phylogenetic analysis revealed that *P. nitida* is closely related to *Paris fargesii* and forms a cluster with *Paris dunniana*. These findings provide significant genomic information for further research on the evolutionary, taxonomic, and phylogenetic aspects of the genus *Paris*.

## Introduction

Species within the genus *Paris* (Melanthiaceae) are perennial herbs widely distributed across temperate and subtropical regions of Asia, particularly in China, where they hold significant medicinal value (Zhang et al. 2015; Liu et al. 2016; Xie et al. 2017). Due to habitat fragmentation, overharvesting, and limited natural regeneration, all species within the *Paris* genus are classified as critically endangered because of their restricted wild populations (National Forestry and Grassland Administration, 2021). *Paris nitida* G.W.Hu, Z.Wang & Q.F.Wang 2017, a newly discovered species in 2017 (Wang et al. 2017), is found in subtropical evergreen forests in the Hunan and Hubei provinces of China. The plants exhibit subcoriaceous, lustrous leaves and solitary flowers consisting of two whorls of tepals. The outer whorl comprises leaf-like sepals, while the inner whorls consist of linear petals. Notably, the wild populations of *P. nitida* are particularly small and are designated as grade II in the Information System of Chinese Rare and Endangered Plants (ISCREP) (http://www.iplant.cn/rep). In recent years, genetic information derived from chloroplast genomes has been extensively utilized to explore plant phylogenetic relationships due to its maternal inheritance and relatively compact size. The chloroplast genomes of various *Paris* species have been sequenced for phylogenetic analysis prompting a reevaluation of the genus’s classification and resolving phylogenetic ambiguities (Song et al. 2017; Song et al. 2019; Wang et al. 2019; Zhao et al. 2019; Fan et al. 2020; Jiang et al. 2021; Ling and Zhang 2020; Zhang et al. 2021). However, the complete chloroplast genome of *P. nitida* has yet to be sequenced. In this study, we employed the Illumina high-throughput sequencing technique to obtain the chloroplast genome of *P. nitida* and conducted a comparative analysis with other species within the *Paris* genus. This research represents the first investigation into the features of the chloroplast genome sequence of *P. nitida* and aims to ascertain its phylogenetic position. Our primary objective is to provide a valuable genomic resource for *P. nitida*.

## Materials and methods

**Figure 1.**
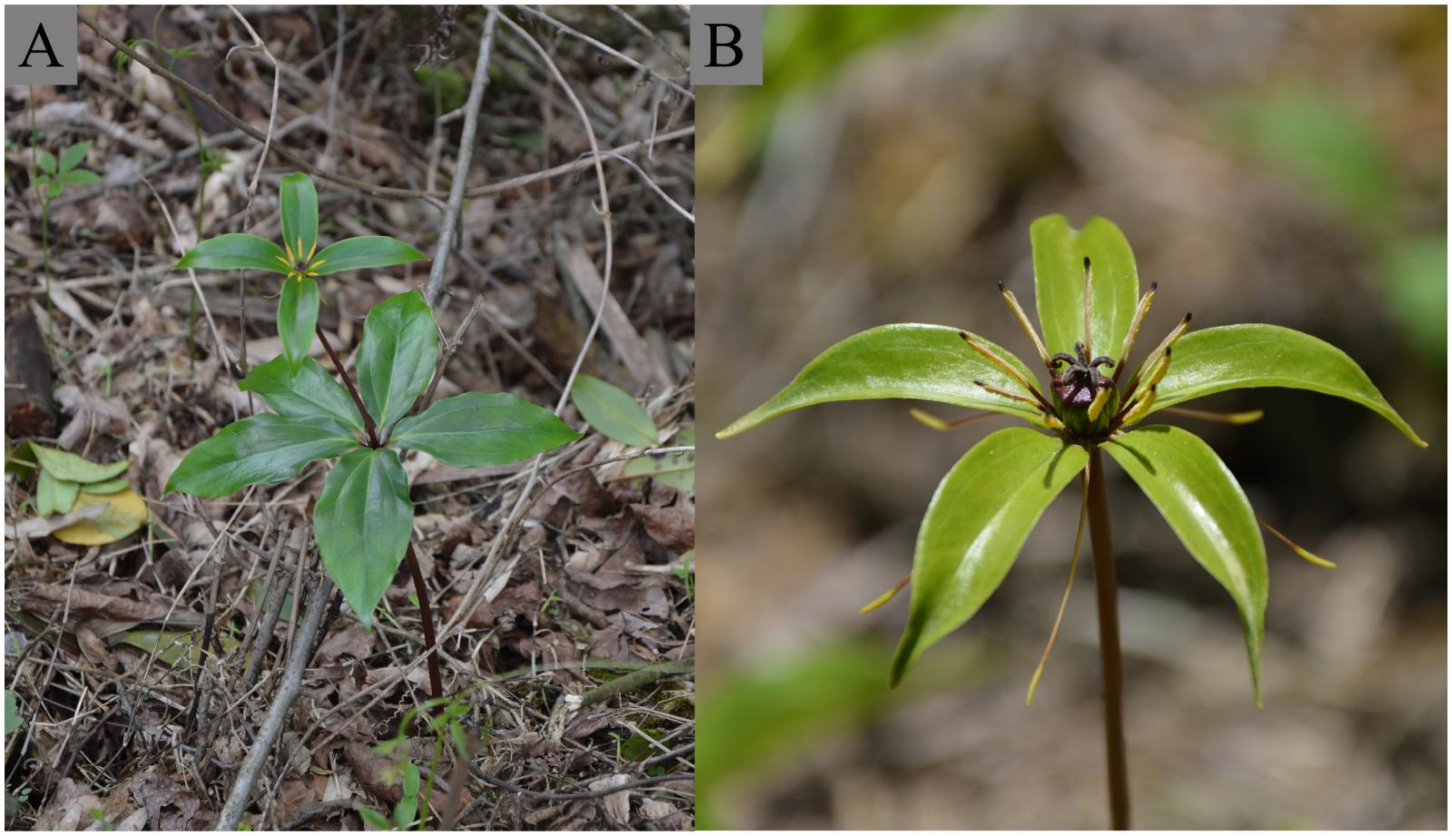
Pictures of *P. nitida* (voucher specimen number YHH_2023_LiDu_HAF). (A) Habit, (B) Flower. All photos were taken by Prof. Xujun Wang from Liulin, Dujiaping, Yuanling, Huaihua, Hunan, P. R. China (28°27′33.58″ N, 110°53′23.95″ E).

Plants of *P. nitida* were found to grow in Liulin, Dujiaping, Yuanling, Huaihua, Hunan, P. R. China (28°27′33.58″ N, 110°53′23.95″ E; altitude: 581 m) (Figure 1). The harvested leaves were rinsed three times with sterile deionized water, wrapped in tinfoil, and then snap-frozen in a dry ice-ethanol bath. A voucher specimen was retained in a freezer at −70 °C with the voucher specimen number YHH_2023_LiDu_HAF at Hunan Academy of Forestry (Contact person: Prof. Xujun Wang, xjwang0514@sina.com).

We purified total genomic DNA from 200 to 300 mg of leaf tissue using the CTAB protocol (Doyle and Doyle, 1987). The quality and purity of the isolated genomic DNA were evaluated through 1.2% agarose gel electrophoresis and spectrophotometry using a NanoDrop 8000 spectrometer (Thermo Scientific, Waltham, MA, USA),. For library construction, 200 ng of the qualified genomic DNA was randomly fragmented to an average size of 350 bp by an ultrasonicator Covaris S-series (Covaris, Woburn, MA, USA). A final paired-end DNA sequencing library was prepared following a series of steps, including DNA purification, end repair, A-tailing, adapter ligation and PCR amplification. Subsequently, the sequencing was performed using an Illumina HiSeq X platform (Illumina, San Diego, CA, USA) at Changsha Aoji Biotechnology Co., Ltd., Changsha, China.

In total, we obtained 76,790,170 raw reads and 76,203,160 clean paired-end reads. We performed a *de novo* assembly of the chloroplast genome of *P. nitida* using SPAdes software version 3.11.1 (Bankevich et al. 2012), in comparison to the reference chloroplast genome of *Paris polyphylla* var. *Chinensis* (GenBank: MN528722). The assembly was annotated using the CPGAVAS2 (Shi et al. 2019) and DOGMA (Wyman et al. 2004) software with default parameters. We retrieved ten complete chloroplast genome sequences from the NCBI GenBank database, aligned these sequences along with the chloroplast genome of *P. nitida* using the MAFFT v7.313 (Katoh and Standley, 2013) plugin integrated into PhyloSuite v1.2.1(Zhang D et al. 2020). A maximum likelihood phylogenetic tree was reconstructed utilizing IQ-TREE v1.6.8 (Nguyen et al. 2015), employing the GTR + F + I + G4 nucleotide substitution model and 5000 ultrafast bootstraps. Finally, FigTree v1.4.4 was used for the visualization of the resulting phylogenetic tree.

## Results

**Figure 2.**
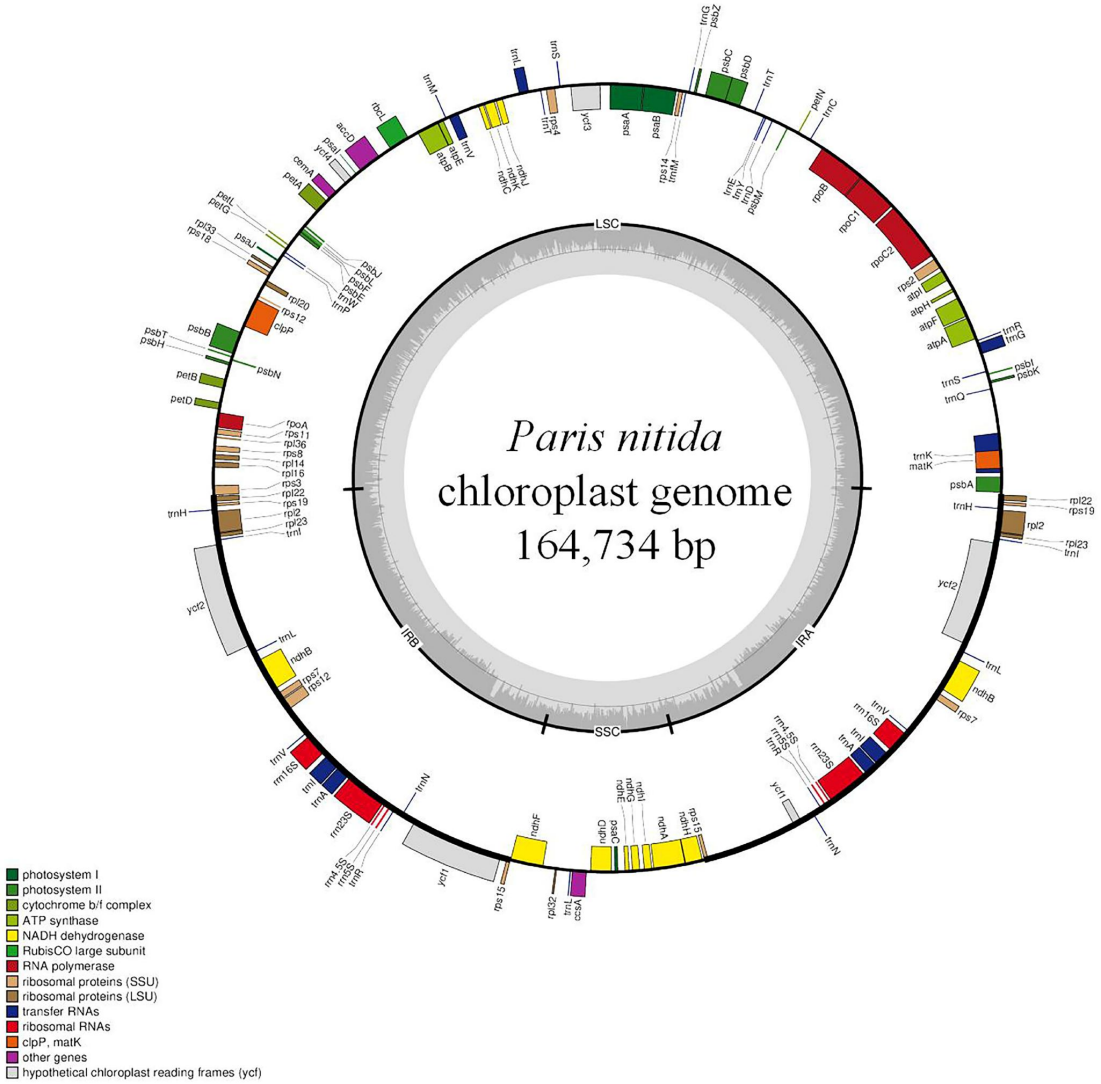
Circular representation of the chloroplast genome of *P. nitida*. The genes depicted both inside and outside the circle are transcribed in a counterclockwise and clockwise direction, respectively. Various colors illustrate genes categorized by their functions. The dashed area in the inner circle displays the GC and AT content, represented by dark and light grey, respectively. Abbreviations: LSC refers to the large single-copy region; IR denotes the inverted repeat; and SSC indicates the small single-copy region.

The average, maximal and minimal depths of read coverage for the assembly of the *P. nitida* chloroplast genome were found to be 1,661×, 3,328× and 17×, respectively (Figure S1). The final chloroplast genome obtained (GenBank: OP425116) consists of 164,734 bp, with a total AT content of 63.12% (Figure 2). Its structure is characterized by a typical quadripartite arrangement, which includes a large single-copy region of 93,593 bp and a small single-copy region of 18,429 bp, exhibiting AT contents of 62.89% and 59.70%, respectively. Two inverted repeat segments, each measuring 26,356 bp and having an AT content of 64.83%, separate the aforementioned single-copy regions into the small and large single-copy regions.

A total of 131 genes have been annotated, which includes 36 genes for tRNA, 87 coding for proteins, and eight for rRNA. Notably, 20 of these genes contain introns, comprising 16 genes (*trnA-UGC*, *trnI*-*GAU*, *ndhB*, *rpl2*, *trnK*-*UUU*, *trnG*-*GCC*, *atpF*, *rpoC1*, *trnL*-*UAA*, *trnV*-*GAC*, *accD*, *rpl2*, *ndhB*, *trnI*-*GAU*_copy2, *trnA*-*UGC*_copy2, *ndhA*), each harboring a single intron; two genes (*ycf3* and *clpP*), each harboring two introns, and two genes (*ycf2* and *ycf2*_copy2), each harboring three introns (Figure S2). Interestingly, among the annotated genes, the *rps12* gene is characterized as a trans-spliced gene, featuring a single exon at the 5′-end located in the large single-copy region, while the duplicated exons at the 3′-end are situated in the inverted repeat regions (Figure S3).

**Figure 3.**
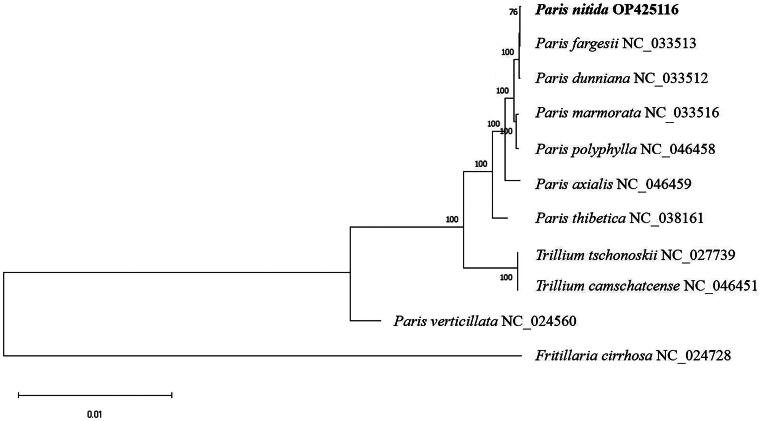
Phylogenetic tree based on complete chloroplast genome sequences of 11 species. The following sequences were used: *P. nitida* OP425116 (this study), *P. fargesii* NC_033513 (unpublished), *P. dunniana* NC_033512 (unpublished), *P. marmorata* NC_033516 (Gao et al. 2018), *P. polyphylla* NC_046458, *P. axialis* NC_046459 (unpublished), *P. thibetica* NC_038161, *trillium tschonoskii* NC_027739 (unpublished), *T. camschatcense* NC_046451 (Guan et al. 2021), *P. verticillata* NC_024560 (Gao et al. 2018), *Fritillaria cirrhosa* NC_024728 (Li et al. 2015). Supports for nodes were calculated *via* 1000 standard bootstrap replicates. Scale bar = 0.01.

To further evaluate the phylogenetic placement of *P. nitida*, complete chloroplast genome sequences of eight *Paris*, two *Trillium* and one *Fritillaria* species were subjected to phylogenetic analysis. The two *Trillium* and one *Fritillaria* species were chosen as outgroups. The results revealed that *P. nitida* is sister to *P. fargesii*, and together, they are sister to *Paris dunniana.* This clade as a whole is sister to *Paris marmorata* and *Paris polyphylla* (Figure 3).

## Discussion and conclusion

This investigation presents the first complete chloroplast genome sequences with gene annotations for *P. nitida*, a new species identified during field surveys. Our analysis revealed that the chloroplast genome of *P. nitida* exhibits high similarities with other species within the genus regarding genome structure, size, AT content, gene number and gene diversity (Song et al. 2017; Song et al. 2019; Wang et al. 2019; Zhao et al. 2019; Fan et al. 2020; Jiang et al. 2022; Ling and Zhang 2020; Zhang et al. 2021). Phylogenetic reconstruction indicated that both *P. nitida* and *P. fargesii* are phylogenetically closer to *P. dunniana* than to other species within the genus *Paris.* In summary, our findings provide essential genetic data that enhance the phylogenetic classification and evolutionary analysis of the genus *Paris*.

## Supplementary Material

Figure S3.jpg

Figure S2.jpg

Figure S1.jpg

## Data Availability

The associated data that support the findings of this study are openly available in GenBank of NCBI at (https://www.ncbi.nlm.nih.gov/) under accession no. OP425116. The associated BioProject, SRA, and Bio-Sample numbers are PRJNA874521, SRR21290286, and SAMN30551706, respectively.
