# Supplementary figures and images for: The complete chloroplast genome sequence of Paris nitida G.W.Hu, Z.Wang & Q.F.Wang 2017 (Melanthiaceae), an endemic herb in China
Source: Mitochondrial DNA B Resour. 2025 Jun 17;10(7):579–83. doi: 10.1080/23802359.2025.2519214 (PMC12175183; doi:10.1080/23802359.2025.2519214)

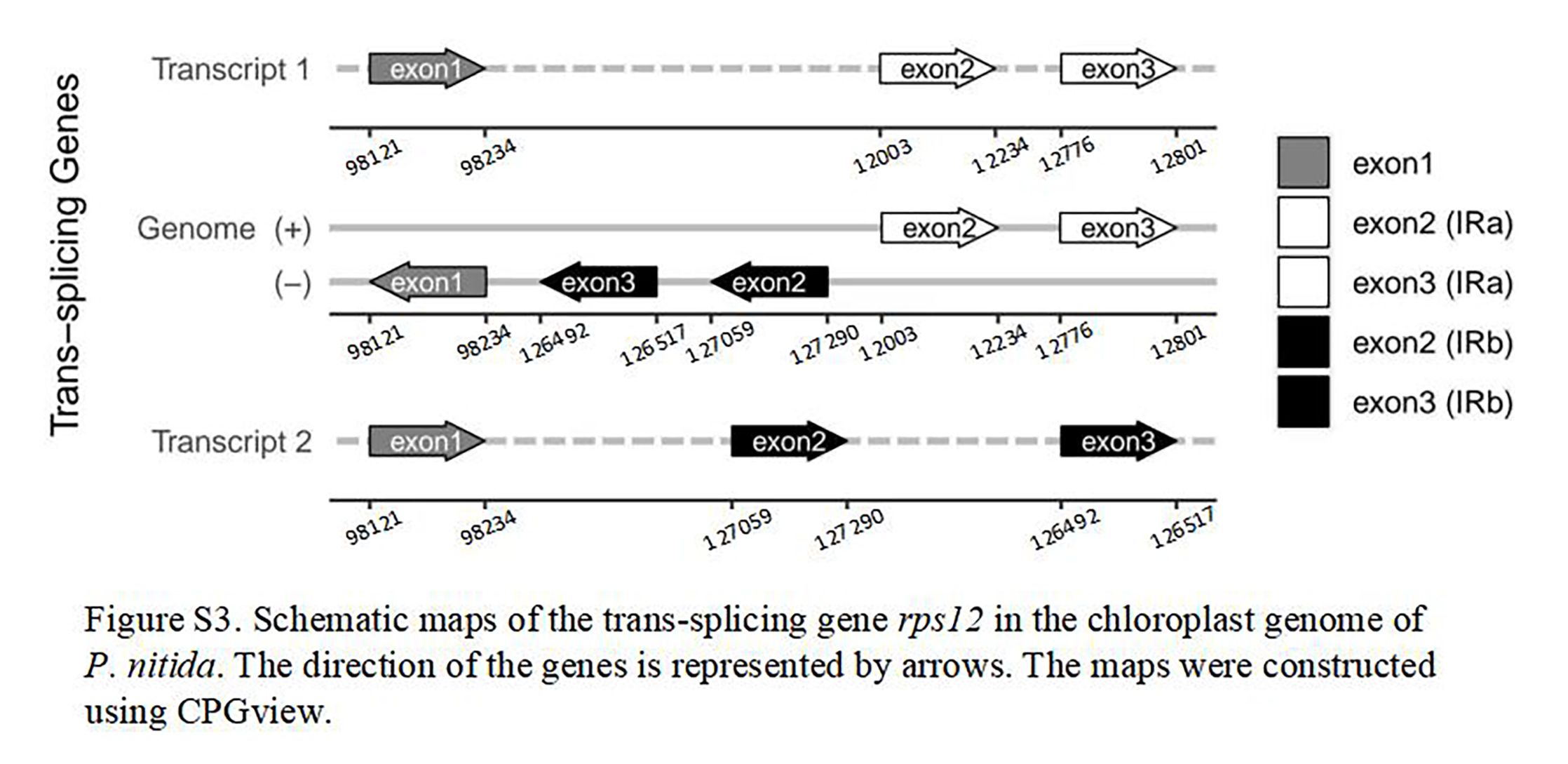

Supplement: Figure S3.jpg [file TMDN_A_2519214_SM5697.jpg]

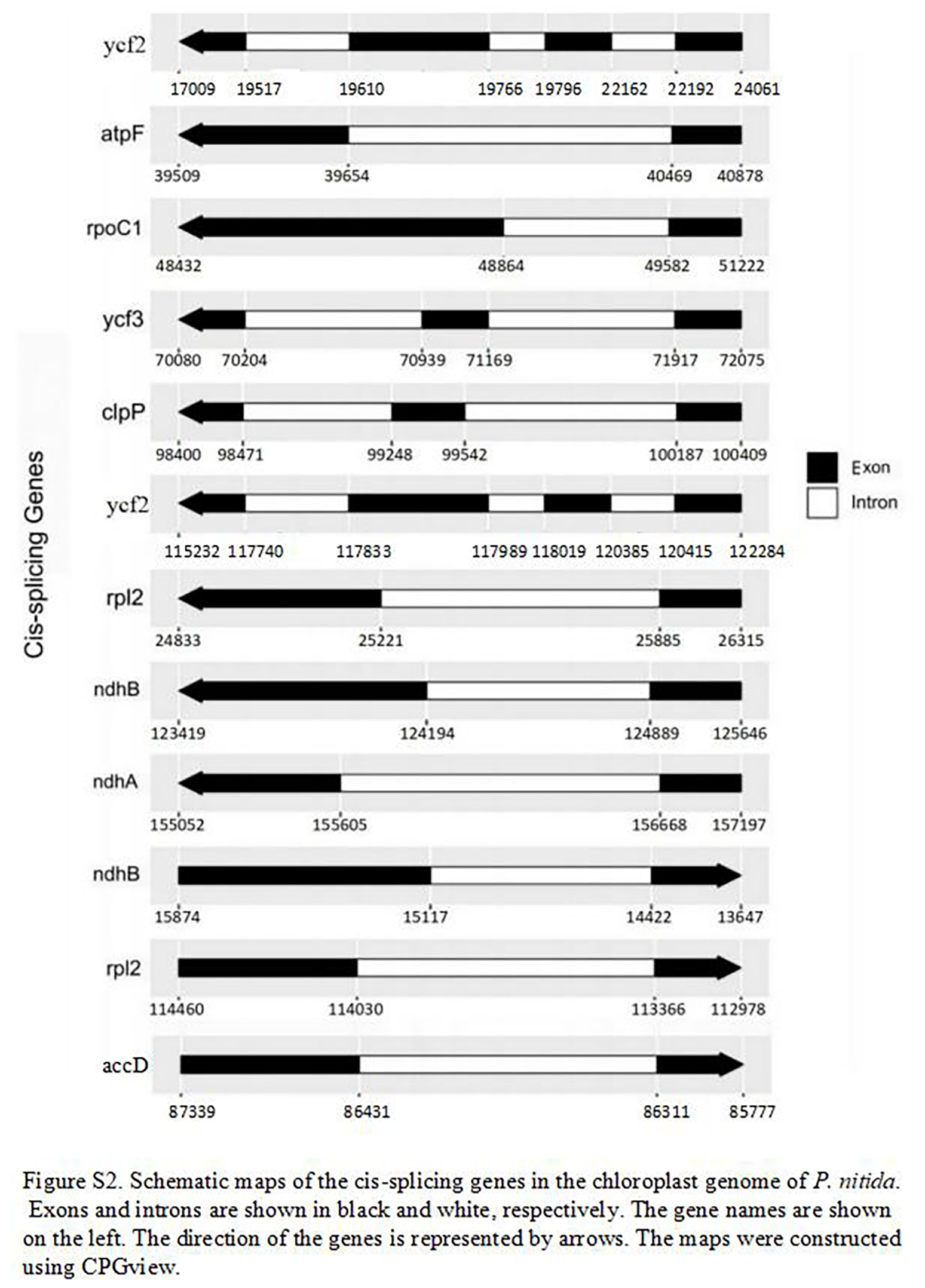

Supplement: Figure S2.jpg [file TMDN_A_2519214_SM5696.jpg]

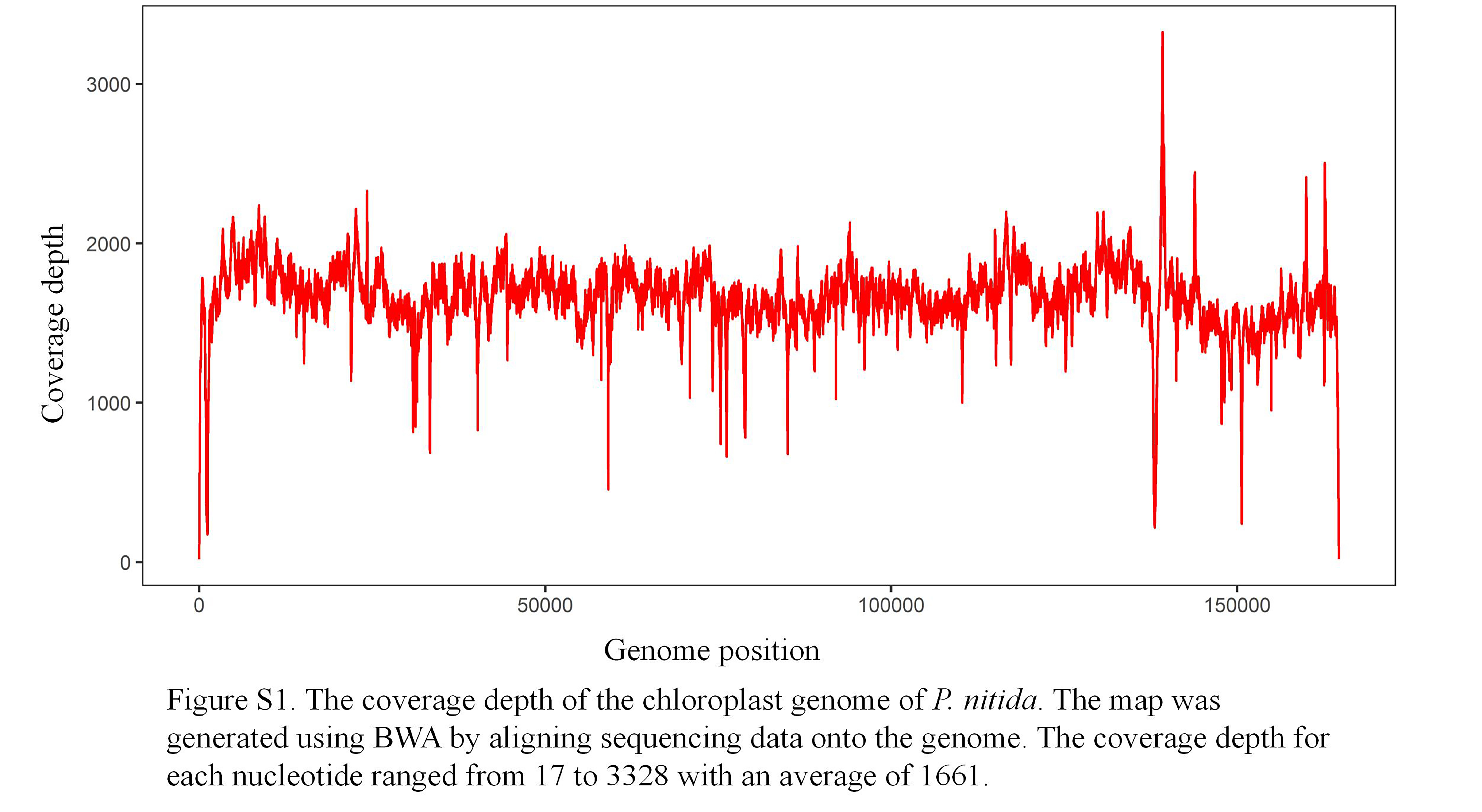

Supplement: Figure S1.jpg [file TMDN_A_2519214_SM5695.jpg]
